# Psychometric Evaluation of Turkish Version of the Cultural Competence Assessment Tool: CCA-EUnurse Project

**DOI:** 10.3390/healthcare11050670

**Published:** 2023-02-24

**Authors:** Aynur Uysal Toraman, Sevcan Topçu, Ebru Konal Korkmaz, Laura Visiers-Jiménez

**Affiliations:** 1 Public Health Nursing Department, Faculty of Nursing, Ege University, 35040 Izmir, Turkey; 2 Campus San Rafael, Escuela Universitaria de Enfermería y Fisioterapia San Juan de Dios, Universidad Pontificia de Comillas, 28036 Madrid, Spain

**Keywords:** cross-cultural instrument adaptation, cultural competence, methodological study, nurses

## Abstract

It is important to understand nurses’ cultural competency all across the world, as globalization and international migration are increasing day by day. The evaluation of the cultural competence of nurses is necessary to provide better quality and adequate health services to individuals, and to improve patient satisfaction and health outcomes. The aim of this study is to evaluate the validity and reliability of the Turkish version of the “Cultural Competence Assessment Tool”. The methodological study was performed to assess instrument adaptation and validity and reliability testing. This study was conducted in a university hospital in western region of Turkey. The study sample consisted of 410 nurses who worked in this hospital. Validity was tested using content validity index, Kendall’s W test and exploratory and confirmatory factor analyses. Reliability was tested using item-total and interitem correlations, Cronbach’s α coefficient of reliability and test–retest analysis. The results of this research demonstrated that the Cultural Competence Assessment Tool showed a good construct validity, internal reliability and test–retest reliability. Confirmatory factor analysis indicated that a construct with four factors showed an acceptable model fit. In conclusion, this study concluded that the Turkish version of the Cultural Competence Assessment Tool is a valid and reliable measurement tool.

## 1. Introduction

Globalization and international migrations cause cultural diversity in societies, which consequently result in multicultural population structures all over the world, where individuals, families and groups from different cultures come together [1,2,3]. This situation causes health care professionals to interact with individuals, families and groups with different health–disease perceptions, beliefs, languages and life experiences. However, health care initiatives that are not adequately grounded within the context of cultural differences result in inadequate health care services [4]. For this reason, it is very important for health professionals to gain cultural competence in order to provide culturally adequate care services [5].

The concept of cultural competence was firstly introduced in the 1980s and nurse Madeline Leininger is the founder of one of the most well-known models in regard to cultural competence [5]. Leininger defined cultural competence as providing respectful care that provides health and well-being along with the strength to face death and disability for individuals and groups from different cultures [6]. Cultural competence refers to the learning about the cultures of different countries and communicating in accordance with the relevant rules along with being conscious of regional cultural differences within the same country and conveying verbal and non-verbal messages in this regard [7].

Health care professionals, in particular nurses, encounter and provide nursing care to individuals from different cultural backgrounds every day. Accordingly, nurses are required to acquire and incorporate cultural competence in order to provide high quality nursing care to individuals [8]. Cultural competence is a componence that plays a key role in providing effective and culturally responsive health care services, alleviating health care inequalities and improving patient satisfaction and health outcomes [5,6,7,8,9]. 

For this reason, there is a need for a measurement tool that will evaluate cultural competencies of health care professionals in a valid and reliable way [10,11]. The Cultural Competence Assessment Tool (CCA) developed by Schim et al. (2003) [12] is widely used in the literature. This study that is an instrument adapted for validity and reliability testing and was developed in the context of the international project Cultural Competences Assessment in Europe (CCA-EU), which principally aimed to assess the evolution of cultural competence in nurses and nursing students from different European universities throughout their undergraduate education, considering both their own and acquired factors. The validity and reliability of the Cultural Competence Assessment Tool (CCA) has already been tested in many different languages [2]. However, a validity and reliability study has not yet been conducted for the Turkish language. An evaluation tool is needed to accurately assess the cultural competence of Turkish nurses. The creation of a valid and reliable tool will facilitate the conduct of relevant studies that will enrich the literature on the cultural competence of nurses. The aim of this study is to evaluate the validity and reliability of the Turkish version of the “Cultural Competence Assessment Tool (CCA-TR)”.

### Conceptual Framework

The concept of cultural competence was first used in the published literature by Cross, Bazron, Dennis and Isaacs [5]. In nursing, the concept of cultural competence stems from Leininger’s studies on transcultural nursing. Leininger developed the sunrise model, which is one of the best-known models of cultural care, and explains the components of her cultural care diversity and universality theory [13]. The sunrise model guides the researcher and clinician in using the theory to provide culturally congruent care to individuals, families, communities and institutions. Leininger defined culturally competent nursing care as “The explicit use of culturally based care and health knowledge that is used in sensitive, creative, and meaningful ways to fit the general lifeways and needs of individuals or groups for beneficial and meaningful health and well-being or to face illness, disabilities, or death” [13].

Cultural competence, which helps guide the delivery of health care to individuals and to populations, is defined as the incorporation of one’s cultural diversity experience (fact), awareness (knowledge) and sensitivity (attitude) into everyday practice behaviors [14]. The cultural competence model, which has four elements, including cultural diversity, cultural awareness, cultural sensitivity, and cultural competence, was proposed by Schim and Miller [15]. Schim et al. (2003) [12] developed the CCA tool to measure cultural competence based on Schim and Miller’s (1999) Cultural Competence Model. Cultural diversity, also called cultural plurality, is a fact of life in health care settings [15]. Cultural diversity means that various cultural patterns coexist in a given geographical area. Cultural awareness relates to health care professionals’ knowledge of differences and similarities in cultural expression. Cultural awareness does not only evaluate differences in terms of language, religion and food knowledge but also defines how these areas affect minority people’s approaches to care. Cultural sensitivity requires recognizing that culturally based values, beliefs and practices influence people’s health and lifestyles and need to be considered in plans for service. Cultural sensitivity means that culturally based values, beliefs and practices affect people’s health and lifestyles. The nursing care of vulnerable people is shaped within the framework of cultural competence, social justice and human rights [12,15].

## 2. Material and Methods

### 2.1. Design and Sampling

This methodological study was conducted to test the validity and reliability of the Cultural Competences Assessment Tool in Turkey. This study was conducted in a university hospital in western region of Turkey between July to August 2021. The University Hospital became operational in 1964 as a research and training hospital and has a capacity of 1806 beds for patients. Approximately 1494 doctors, 1567 nurses and 676 other health care personnel in various units work at University Hospital. University Hospital provides health care services in two emergency services, 22 intensive care units, 16 operating rooms, 34 policlinics and 210 laboratories. As the largest hospital in the region, it serves approximately one million patients annually.

The sample size was estimated based on the criterion that at least 10 participants per item were required for conducting an exploratory and confirmatory factor analysis of an instrument. In addition, the sample size was estimated based on the G Power package. The close fit and not-close fit were tested in G Power, and the power exceeded 0.99 in both instances. The sample size was between 100~150 [16]. Thus, a sample of 410 for EFA and CFA was determined to have adequate power to detect effects. The inclusion criteria of the study were nurses who work in University Hospital and who agreed to complete the questionnaire. The 410 nurses who voluntarily consented to participate in the research. Data were collected through face-to-face interviews with volunteer nurses. The performance of the interview took an average of 5 to 8 min.

### 2.2. Data Collection

Data collection took place between July and August 2021 at University Hospital. The questionnaire was distributed by researchers to 460 nurses who work in 41 units. There were 50 participants who did not answer some of the questions and were, therefore, excluded from the survey. The remaining 410 (89.1%) nurses completed the self-administered survey in full and were included in this study. 

### 2.3. Instrument

Research data were collected using Descriptive Information Form and Cultural Competence Assessment Instrument (CCA). The Descriptive Information Form consists of seven items: age, gender, marital status, educational status, working time as a nurse and feeling competent while working with individuals from different cultures.

CCA was developed based on the Cultural Competence Model articulated by Schim and Miller (1999) [15]. The CCA is designed to measure cultural diversity experience, cultural awareness and sensitivity and cultural competence behaviors. The CCA was originally developed by Schim et al. (2003) [12]. It has been translated and validated into Italian (CCAI-25), Korean (KCCA-16) and Spanish (CCA-S) [2,10,17]. CCA has been used many times to evaluate the cultural competencies of health care professionals, such as nursing students, nurse practitioners and nurses [17,18,19]. CCA consists of 25 items and two sub-scales of the Cultural Competence Behaviors and Cultural Awareness and Sensitivity. The 11-item Cultural Awareness and Sensitivity subscale measures cultural awareness and sensitivity. The Cultural Awareness and Sensitivity subscale uses a 7-point Likert scale (7 = strongly agree to 1 = strongly disagree) and has a no opinion option. The 14-item Cultural Competence Behaviors subscale measures cultural competence behaviors. The Cultural Competence Behaviors subscale uses a 7-point Likert scale (7 = always to 1 = never) and has a not sure option. Summing items from the Cultural Awareness and Sensitivity and Cultural Competence Behaviors subscales yields the subscale scores. Four items on the Cultural Awareness and Sensitivity subscale are negatively phrased and are reverse-scored for data analysis. Higher scores indicate higher levels of knowledge, more positive attitudes and a greater frequency of competence behaviors. Cronbach’s α value for CCA was 0.91 [12].

### 2.4. Validation Process of the Scale

In translation adaptations, the original structure should be used, and items that do not fully conform to the target culture should be changed. After obtaining permission from S. Schim, who developed the CCA, the scale was translated into Turkish by two language experts. Then, the Turkish version was adjusted by the researcher, and after appropriate revisions were made, the questionnaire took its final form. The Turkish version of the scale was called the Cultural Competence Assessment Turkish Version (CCA-TR).

After obtaining authorization to use the CCA from the developer, a native Turkish nursing professor translated the questionnaire from English to Turkish. A nursing professor and a Turkish language teacher reviewed the translated questionnaire for incomprehensible or ambiguous wording and cultural appropriateness. The Turkish version was then back-translated to English by a bilingual Turkish teacher. The reverse translation was compared to the original version by the instrument’s developer. She confirmed that the CCA had been translated accurately and that there was no change in the instrument’s meaning due to the translation process. Standard procedures were followed in order to verify the accuracy of translation and scope of the Turkish version of the scale by eight experts. The opinions of eight experts who are professionals in the fields of sociology, nursing, Turkish language and literature and who study culture and cultural competence were sought. The experts were asked to assess the items on the following scale: inappropriate (1 point), needs serious review (2 points), needs slight revision (3 points) and appropriate (4 points). Content validity index (CVI) was calculated by dividing the number of experts who had a rating of either 3 or 4 by the total number of experts. Based on their responses, the CVI and content validity ratios (CVR) were calculated. If the CVI and CVR were more than 0.80, then it was interpreted as being indicative of a high content validity [16,20]. The construct validity was evaluated using both exploratory (EFA) and confirmatory (CFA) factor analyses. EFA was used to determine the relationship between the items and factors. The Kaiser–Meyer–Olkin (KMO) and Bartlett test were used to assess whether the sample was suitable for factor analysis. Being greater than 0.50 for the KMO value is appropriate and Bartlett’s test of sphericity requires *p* < 0.001 to be acceptable for factor analysis [20]. Validity was tested using EFA and CFA. Factors were extracted on the basis of the results of a scree plot, eigenvalues, total variance and conceptual considerations. Factor loading of 0.40 or greater was used to identify items contributing to a given factor [21]. In multifactor designs, it is considered sufficient to have the explained variance be between 40% and 60% [22] The CFA tested the model fit of the extracted factor model. Multiple model fit indices, including χ^2^/degrees of freedom (χ^2^/sd) (less than 5), root mean square error of approximation (RMSEA) (excellent ≤ 0.05; good ≤ 0.08), goodness-of-fit index (GFI) (excellent ≥ 0.95; good ≥ 0.90), comparative fit index (CFI) (excellent ≥ 0.95; good ≥ 0.90), Tucker–Lewis index (TLI, excellent ≥ 0.95; good ≥ 0.90) and normal fit index (NFI, excellent ≥ 0.95; good ≥ 0.90), were also used [23,24,25,26,27].

### 2.5. Reliability

The internal consistency, test–retest reliability, intraclass correlation coefficient and paired-sample *t*-tests were examined. Internal consistency was further evaluated with item-total correlations and interitem correlations. Item-total and interitem correlations were evaluated to examine the homogeneity of the CCA-TR. An acceptable coefficient for item-total correlations is greater than 0.40 [22]. To determine item discriminating power, revealing the item discrimination between the groups, a corresponding test–retest was conducted. In addition, the item discrimination powers were investigated by examining the *t*-values, taking into account the difference between the highest 27% and the lowest 27% groups. Moreover, the discriminating index, expressed by a D-value, was calculated by subtracting the percentage of correct answers in the worst-performing group from the percentage of correct answers in the best-performing group [28,29]. To specify the unchangeability against time (test–retest method) of CCA-TR, the scale was reapplied to 30 individuals two weeks after the first application of parametric statistical testing [29]. For reliability, test–retest reliability, intraclass correlation coefficient and paired sample *t*-tests were used. The correlation obtained in determining the scale’s consistency over time should be positive, and it should indicate a high correlation and be at least greater than 0.70 [30].

### 2.6. Analysis

The Statistical Package for the Social Sciences (SPSS) 22.0 and the Analysis of Moment Structures (Amos) 21.0 statistics package programs were used in the analysis of the data analysis. Information on the introductory characteristics of the individuals involved in the sample was analyzed using the number and percentage distribution. The significance level was considered as *p* < 0.05 in the validity and reliability analysis.

### 2.7. Ethical Considerations

To conduct the study, permission was obtained from the Ege University Scientific Research and Publication Ethics Committee (Approval Number: 06/10-1007). Permission was obtained from the developer of the original scale. Before data collection, participants were informed about the research objectives and procedures, and their written permission was obtained via an informed consent form. All participants were informed that their participation in the study was entirely voluntary.

## 3. Results

### 3.1. Participants

Mean age of the nurses participating in the study was 35.30 ± 7.33; 92.4% were women and 83.2% of them were university graduates. A total of 54.1% of the nurses declared that they had provided nursing care to a patient from a different culture in the previous year and the average number of patients from different cultures who were provided nursing care was 4.54 ± 11.42. A total of 55.3% of the nurses evaluated their ability to provide nursing care for individuals from different cultures as “sufficient”, 31.7% as “partially sufficient” and 13% as “insufficient”.

### 3.2. Validity Analysis

In line with reviewers’ opinions, items 5, 7, 9, 12, 13, 16–19, 21 and 25 were revised and rephrased. The content validity ratios (CVR) of the items were found to range between 0.75–1.00, whereas the content validity index (CVI) was determined as 0.94. Kendall’s W test revealed that the level of agreement between the reviewers were statistically acceptable (Kendall’s W = 0.344, *p* = 0.000).

The KMO value of 0.857 and χ2 value of 8314.26 (*p* < 0.001), which were further interpreted as the sample for factor analyses.

**Figure 1:**
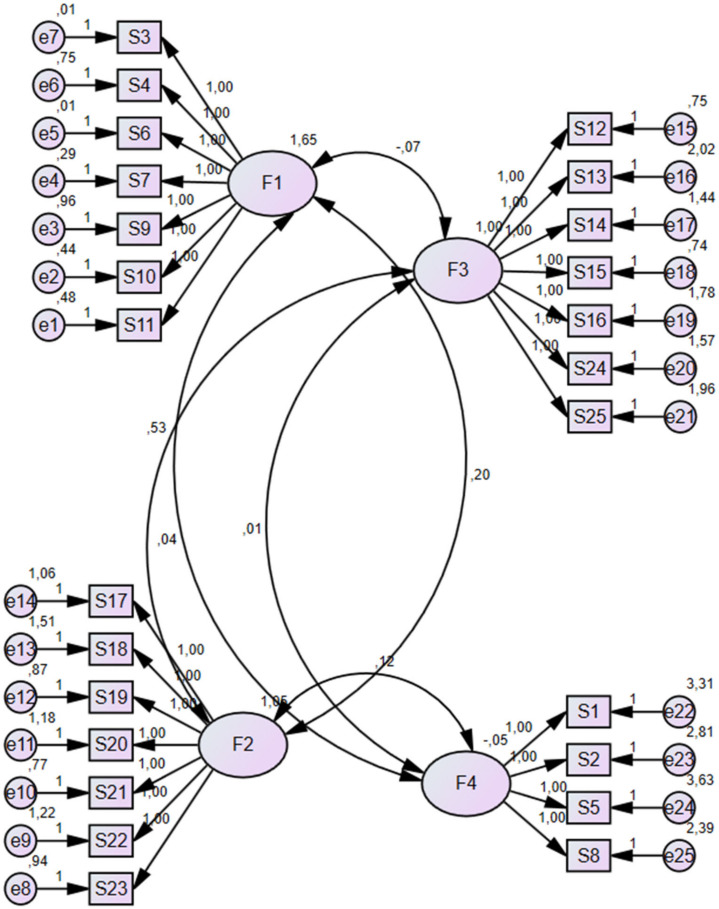
Structural equation model of the CCA-TR.

According to the principal components analysis, there were four factors with an eigenvalue greater than 1. The eigenvalues of the four factors were factor 1 = 6.19, factor 2 = 5.14, factor 3 = 1.99 and factor 4 = 1.50. These factors explained 59.30% of the total variance. The first, second, third and fourth factors, respectively, explained 23.14%, 16.71%, 13.31% and 6.14% of the total variance. The results of the Varimax Rotation Procedure indicated that factor load values for 25 items of the scale ranged between 0.47 and 0.98 (Table 1). Confirmatory factor analyses (CFA) were performed in the second part of the CCA-TR (25 questions). CFA was performed in order to explore the structural validity of the CCA-TR. The model of this study was equivalent to the original factorial structure of the CCA-TR scale, as recommended by the constructors. The four-factor model was performed through 25 questions and a demonstrated a satisfactory fit to the Turkish nurses’ sample. The measurement model (Figure 1) was tested for confirmatory factor analysis, and it indicated a good fit with the indices (χ2/df = 3.48; RMSEA = 0.061; CFI = 0.94; NFI = 0.95; GFI = 0.93; TLI = 0.95; IFI = 0.93). Moreover, a positive correlation was seen between all the subscales of the CCA-TR (*p* < 0.0019). The CFA confirmed the four-factor structure of the scale, and the final instrument included 25 items: “Cultural Awareness” (7 items), “Active Behavior” (7 items), “Seeking Information” (7 items) and “Sensitivity” (4 items). The four factors for the 25 items of the CCA-TR can be seen in Figure 1.

### 3.3. Reliability Analysis

Item analysis revealed that the item-total correlation coefficients of the items ranged between 0.44 and 0.70. T-test results conducted for calculating item discriminating power indicated that each item had the power to statistically significantly discriminate the difference between the highest 27% and the lowest 27% groups of the sample (*p* < 0.001). The item difficulty indices of the instrument varied between 0.21 and 0.88 and discrimination values (D-values) were between 0.20 and 0.70. Cronbach Alpha value of the scale is 0.83, whereas Cronbach Alpha coefficients of the sub-dimensions ranged between 0.80 and 0.96. Results of Pearson product-moment correlation analysis performed to determine time invariance (test–retest correlation) indicated a strong positive correlation between mean total test scores of the nurses in the first application of the test and mean total scores of the nurses obtained after the test is re-applied two weeks later (*p* < 0.001). The Cronbach Alpha values of the scale and the test–retest analysis results are given in Table 2.

## 4. Discussion

To assure language validity, the procedures recommended by the WHO (2019) [31] and the International Test Commission (ITC) [32] for the adaptation of tools to foreign languages were used. In the framework of these recommended approaches, a content validity study was performed [16]. This study is the first attempt to translate a full version of scale into Turkish and to test its psychometric aspects. The panel of experts (*n* = 8) reviewed the CCA-TR, which resulted in high scores on equivalence, clarity and readability. The CVI and CVR values with regard to the Turkish form submitted for expert opinion were found to be within the recommended reference values, which indicated that the items of the scale adequately represented the features to be measured and the content validity index of the scale is high. W values between 0.30–0.50 derived as a result of Kendall’s coefficient of concordance (W) test, which was conducted to examine the agreement between reviewers’ opinions, referred to an average effect size [33]. Statistical analysis performed in accordance with the reviewers’ assessments indicated that the reviewers’ opinions were moderately compatible (*p* < 0.001).

The Kaiser–Meyer–Olkin (KMO) measure of sampling adequacy and Bartlett’s test of sphericity analysis were used to determine whether the sample size was sufficient for factor analysis. The KMO coefficient values of 0.5 and above are acceptable. The KMO coefficient is classified in the literature as “excellent” between 0.90–1.00, “very good” between 0.80–0.89, “good” between 0.70–0.79, “moderate” between 0.60–0.69, “poor” between 0.50–0.59 and “unacceptable” below 0.50 [34,35].

Factors with an eigenvalue greater than 1 are considered significant within the scope of factor analysis, whereas factors with eigenvalue less than 1 are not considered [34]. Factors with an eigenvalue greater than 1 were considered in this study. In multifactorial designs, explained variance between 40% and 60% is considered as sufficient [22,28]. A total variance above 40% is in line with the rate stipulated in the literature.

Factor loading is expressed with values between 0–1 [22]. In this study, items with a factor loading of at least “0.40” are considered and each dimension is attributed at least three variables (items) (Table 1). The scale used in the study by Schim et al. (2003) [12] has a two-factor structure. Unlike the original study, the item distribution herein has a four-factor structure. The first factor was defined as “cultural awareness”, the second factor as “attitude”, the third factor as “knowledge” and the fourth factor was defined as “cultural sensitivity”. In case the items in the measurement tools are not suitable for the adapted culture or if they have a different meaning in the adapted culture, the scale structure may be amended [36]. The difference in item distributions in this study is attributed to cultural differences.

The model derived with exploratory factor analysis (EFA) is further evaluated using confirmatory factor analysis (CFA). Regarding goodness-of-fit indices, degrees of freedom less than five in a Chi-Square Test (χ2/sd); RMSEA less than 0.10; GFI below 0.75; CFI above 0.85; and CFI, NFI, GFI, TLI and IFI values above 0.90 indicate a good fit [23,24,25,26,27]. The model, which was re-defined in Turkish, was concluded to validly measure the intended structure with its four sub-dimensions.

Item analysis states that items with an item-total correlation of 0.30 and above are able to appropriately discriminate individuals and items with an item-total correlation between 0.20–0.30 can be included in the scale or should be corrected when necessary, whereas items with an item-total correlation of less than 0.20 should not be included in the scale [28]. The results indicate that the items of the scale bear the highest level of qualifications to be measured and successfully distinguish the measured characteristics of individuals [28,29]. Item discriminating power was supported in all items, indicating that the overall score significantly discriminated between the group in the upper 27% and the lower 27% of the sample [29]. Discrimination is important because if the test items can discriminate more, they will be more reliable. The discrimination and item difficulty values were satisfactory. Cronbach’s alpha coefficients of the scale used in the study and the sub-dimensions were found to be over 0.80 (Table 2). These results indicated that the items were internally consistent with each other and that the internal consistency of the scale was sufficient [37].

Test–retest is one of the best ways to prove invariance in scale studies. The lack of a statistically significant difference, as well as a strong relationship between the test–retest scores, is accepted as proof of invariance [29]. According to paired sample *t*-test results, the first and second application of the test to determine the time invariance of the scale indicated a strong, very significant and positive correlation between sub-scale total scores and scale total score (*p* < 0.001). These findings demonstrated the results’ long-term durability.

### Limitations

The study sample consisted of nurses who worked a university hospital in the western region of Turkey, and it is thought that this may reduce representativeness and limit the generalizability of the results. In Turkey, this scale will be the first to evaluate cultural competence and its four sub-dimensions. Adapting this scale will also help create strategies to improve multicultural care and allow practitioners to provide beneficial care that respects an individual’s culture. Future researchers should recruit a larger sample size of nurses and explore the differences and relationships across culture and social demography characteristics. Additionally, the CCA tool is a self-reported questionnaire. This can lead to social desirability bias in respondents. Parallel-form or alternate-form reliability was not reviewed in this study; further research is needed to estimate the parallel/alternate-form reliability.

## 5. Conclusions

The psychometric properties in the Turkish version of the CCA-TR scale confirmed the validity and reliability of the 25-item scale. It was found that factor structure of the CCA-TR was different to the original version, which is composed of two dimensions, whereas our data seemed to better fit four factors. Cronbach’s alpha coefficients of both the overall scale and sub-dimensions were high, and the Turkish version of the scale achieved cultural equivalence. The study determined that the Turkish version of the CCA is a valid and reliable instrument when used to measure cultural competence for nurses.

## Figures and Tables

**Table 1:** Items and their standardized coefficients on the CCA-TR dimensions.

	Cultural Awareness	Active Behavior	Seeking Information	Sensitivity
Spirituality and religious beliefs are important aspects of many cultural groups.	0.980			
Many aspects of culture influence health and health care.	0.978			
Individual people may identify with more than one cultural group.	0.917			
I think that knowing about different cultural groups helps direct my work with individuals, families, groups, and organizations.	0.895			
I understand that people from different cultures may define the concept of “health care” in different ways.	0.890			
Aspects of cultural diversity need to be assessed for each individual, group, and organization.	0.820			
I believe that everyone should be treated with respect no matter what their cultural heritage.	0.808			
I remove obstacles for people of different cultures when people identify barriers to me.		0.867		
I welcome feedback from clients about how I relate to people from different cultures.		0.826		
I ask people to tell me about their expectations for health services.		0.775		
I remove obstacles for people of different cultures when I identify barriers to services.		0.668		
I find ways to adapt my services to individual and group cultural preferences.		0.655		
I recognize potential barriers to service that might be encountered by different people.		0.642		
I avoid using generalizations to stereotype groups of people.		0.485		
I use a variety of sources to learn about the cultural heritage of other people.			0.888	
I include cultural assessment when I do individual or organizational evaluations.			0.875	
I have resource books and other materials available to help me learn about people from different cultures.			0.674	
I seek information on cultural needs when I identify new people in my work or school.			0.505	
I document cultural assessments if I provide direct client services.			0.501	
I ask people to tell me about their own explanations of health and illness.			0.476	
I document the adaptations I make with clients if I provide direct client services			0.473	
Race is the most important factor in determining a person’s culture.				0.679
People with a common cultural background think and act alike.				0.562
If I know about a person’s culture, I don’t need to assess their personal preferences for health services.				0.553
Language barriers are the only difficulties for recent immigrants to the United Sates.				0.537

**Table 2:** Internal consistency and test–retest reliability for the CCA-TR.

Sub-Dimensions	Item Number	Internal Consistency	Test–Retest Reliability	
		Cronbach’s Alpha	r	*p*
Cultural Awareness	7	0.96	0.995	0.000
Active Behavior	7	0.87	0.933	0.000
Seeking information	7	0.80	0.957	0.000
Sensitivity	4	0.87	0.945	0.000
Cultural Competence Assessment Instrument	25	0.83	0.951	0.000

## Data Availability

Data are available upon request.

## Abbreviations

CCA	Cultural Competence Assessment Tool
CCA-TR	Cultural Competence Assessment Tool Turkish Version
